# COVID‐19 severity from Omicron and Delta SARS‐CoV‐2 variants

**DOI:** 10.1111/irv.12982

**Published:** 2022-04-13

**Authors:** Jesse O. Wrenn, Suman B. Pakala, Grant Vestal, Meghan H. Shilts, Hunter M. Brown, Sara M. Bowen, Britton A. Strickland, Timothy Williams, Simon A. Mallal, Ian D. Jones, Jonathan E. Schmitz, Wesley H. Self, Suman R. Das

**Affiliations:** ^1^ Department of Emergency Medicine Vanderbilt University Medical Center Nashville Tennessee USA; ^2^ Division of Infectious Disease, Department of Medicine Vanderbilt University Medical Center Nashville Tennessee USA; ^3^ Department of Pathology Microbiology and Immunology Vanderbilt University Medical Center Nashville Tennessee USA; ^4^ Department of Otolaryngology Vanderbilt University Medical Center Nashville Tennessee USA

**Keywords:** COVID‐19, Delta, Omicron, SARS‐CoV‐2, severity, whole genome sequencing (WGS)

## Abstract

The Omicron variant of SARS‐CoV‐2 achieved worldwide dominance in late 2021. Early work suggests that infections caused by the Omicron variant may be less severe than those caused by the Delta variant. We sought to compare clinical outcomes of infections caused by these two strains, confirmed by whole genome sequencing, over a short period of time, from respiratory samples collected from SARS‐CoV‐2 positive patients at a large medical center. We found that infections caused by the Omicron variant caused significantly less morbidity, including admission to the hospital and requirement for oxygen supplementation, and significantly less mortality than those caused by the Delta variant.

## 1 INTRODUCTION

The COVID‐19 pandemic has caused significant morbidity and mortality worldwide. A new variant that partially escapes the existing vaccines, Omicron, was discovered by genomic surveillance teams in South Africa in November 2021 and was quickly identified as a variant of concern by the WHO.^1^, ^2^, ^3^ Omicron was estimated to have accounted for approximately 95% of daily new infections in the United States by early January 2022.^4^ Rapid exploration of the clinical severity of infections caused by the Omicron variant, as well as future variants, is critical to the targeted public health response to the evolution of the COVID‐19 pandemic. The goal of this project was to compare the clinical severity of COVID‐19 caused by SARS‐CoV‐2 whole genome sequence confirmed Omicron and Delta variants.

## 2 MATERIALS AND METHODS

We compared the clinical severity of Omicron and Delta variants within a cohort of patients who tested positive for SARS‐CoV‐2 during a period of co‐circulation of the Omicron and Delta variants. SARS‐CoV‐2 variants were identified with viral whole genome sequencing, and clinical outcomes were ascertained via systematic capture of clinical data from the electronic medical records. We examined a randomly selected subset (from a combined total‐positive specimen bank) of SARS‐CoV‐2 positive respiratory samples (nucleic acid amplification methods—see below) within the Vanderbilt Health System (Nashville, Tennessee, USA) during two time windows: November 22 to December 9, prior to the transition to Omicron dominance (Delta percent share 88.7% week ending 12/11) in the HHS Region 4 (AL, FL, GA, KY, MS, NC, SC, and TN) and December 27–28, after the transition to Omicron dominance (Omicron percent share 81.5% week ending 12/25).^4^ Samples tested in this laboratory were collected from patients of all ages who presented for care at multiple outpatient and inpatient locations, including drugstore‐based clinics, walk‐in urgent care clinics, primary care clinics, emergency departments, in‐hospital wards, and intensive care units. Samples were reported as positive for SARS‐CoV‐2 according to the fixed parameters of the given nucleic acid amplification test (NAAT), dictated under terms of FDA emergency use authorization. The Vanderbilt Health System employs several COVID‐19 NAATs in parallel, to ensure diagnostic speed and throughput (including the Roche cobas COVID‐19/Influenza assay; the Roche Liat COVID‐19 and COVID‐19/Influenza assays; the Cepeheid GeneXpert COVID‐19/Influenza/RSV assay; the Diasorin Simplexa COVID‐19 assay; and the Hologic Panther COVID‐19 assay).

Residual sample volumes from specimens positive for SARS‐CoV‐2 were transported to our research laboratory for viral whole genome sequencing. In brief, viral RNA was extracted with the QIAGEN QIAamp Viral RNA Mini QIAcube Kit, and sequencing libraries were prepared with the QIAGEN Enhanced QIAseq DIRECT Enhanced SARS‐CoV‐2 kit. Pooled libraries were sequenced on an Illumina NovaSeq instrument with 2 × 150 bp reads. SARS‐CoV‐2 assembly and lineage classification was performed with the Cecret ( https://github.com/UPHL‐BioNGS/Cecret ) SARS‐CoV‐2 workflow (v.2.1.2021117.1), using BWA^5^ for aligning reads, iVar^6^, ^7^ for calling variants and generating the consensus sequences, and NextClade v.1.4.0^8^ and Pangolin v3.1.14^9^, ^10^ for determining viral variant type. The Omicron variants described in this study are the PANGO‐designated lineage B.1.1.529 and the sublineage BA.1, while the Delta variants described in this study are the PANGO‐designated lineage B.1.621.X and the sublineage AY.XX.

The analytical cohort included patients who tested positive for the Delta variant or the Omicron variant during two time periods: November 22 through December 9 (prior to Omicron dominance) and December 27–28 (after Omicron dominance). Demographic and clinical data were obtained from these patients via systematic capture of electronic health record data from all encounters initiated within a date range from 2 days before the positive SARS‐CoV‐2 test through 14 days after the test. Captured data included age, sex, race, ethnicity, vaccination status, chief complaint, vital signs, laboratory results, clinical diagnoses, hospital admissions, hospital length of stay, supplemental oxygen use, invasive mechanical ventilation, hospital discharge disposition, and death. A patient was considered vaccinated if they had one or more administrations of any SARS‐CoV‐2 vaccine documented in our electronic medical record. This includes vaccines administered at our institution, patient reported vaccinations, and those gathered programmatically from several outside sources. Clinical characteristics and outcomes were reported as either counts and percentages or medians and interquartile ranges and compared between patients with Omicron variant versus Delta variant. Comparisons for dichotomous variables and continuous variables were calculated with the chi‐squared test (Fisher's exact test for counts <5) and rank sum test, respectively. For dichotomous variants, a relative risk with 95% confidence intervals (CI) comparing Omicron variant to Delta variant (referent) was calculated.

To further explore the associations between viral variant and disease severity, we next performed logistic regression with the *lrm* function in the R package *rms* (version 6.2‐0),^11^ setting hospitalization and any oxygen use as the dependent variables. In addition to viral variant, age, sex, race, ethnicity, obesity (defined as a body mass index > 30), and SARS‐CoV‐2 vaccination status were a priori selected to be added to the model as independent variables due to their previously established associations with COVID‐19 severity. Age was transformed with restricted cubic splines with five knots with the *rms*::*rcs* function. Due to the high number of missing data in race, ethnicity, and BMI categories, missing data were placed into a category called “Unknown,” and results are presented for each predictor. Race was simplified to Black, White, Asian, Other, or Unknown. Interquartile odds ratios (OR) and their 95% CIs were found with *rms*::*summary.rms*, and *P* values for each independent variable were calculated with the *rms*::*anova* function.

The Vanderbilt Institutional Review Board approved this study (IRB# 200553). Funding was provided by the US Centers for Disease Control and Prevention (CDC), which did not directly participate in the study.

## 3 RESULTS

**FIGURE 1:**
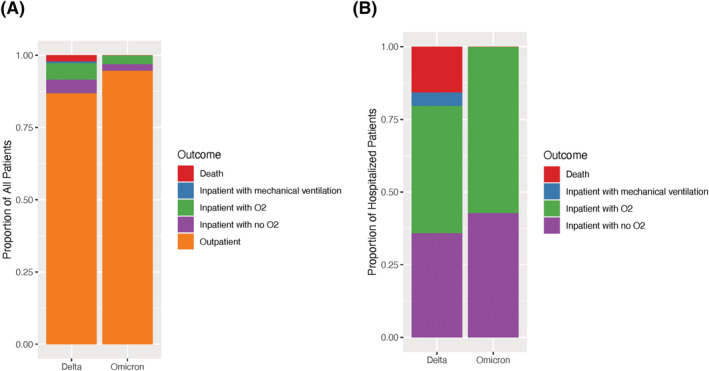
The Delta variant was associated with more severe COVID‐19 than the Omicron variant. (A) The highest severity level experienced on the WHO COVID‐19 Clinical Progression Scale among all patients included in the study, documented in the medical record within 14 days of positive test or during admission initiating within 14 days of positive test, is graphed for patients with Omicron (*n* = 263) vs. Delta (*n* = 489) infection. (B) The highest severity level experienced on the WHO COVID‐19 Clinical Progression Scale is shown for only admitted patients with Omicron (*n* = 14) vs. Delta (*n* = 64) infection. Among patients with Omicron infection, there was no mortality or mechanical ventilation

During the two sample time periods, 23,089 upper respiratory samples underwent SARS‐CoV‐2 RT‐PCR testing at the clinical laboratory at Vanderbilt; 2,688 (12%) were positive for SARS‐CoV‐2. Among the positive samples, 780 (29%) underwent sequencing. Sequencing resulted in 263 (34%) cases identified as Omicron variant, 489 (63%) identified as Delta variant, and 28 (4%) cases in which a lineage was another known variant or not determined. Of the 752 Omicron or Delta whole genomes obtained, 642 with a genome coverage of ≥90% and a passing score on VADR (Viral Annotation DefineR) were published to the Global Initiative on Sharing Avian Influenza Data (GISAID) database. Age, race, and ethnicity were similar for the Omicron and Delta groups; however, there were more females in the Omicron group (Table 1). Several indicators of high disease severity were more common in the Delta group than the Omicron group, including in‐hospital care within 14 days of a positive test (13.1% vs. 5.3%), invasive mechanical ventilation (2% vs. 0%), and death (2% vs. 2%) (Figure 1A,B). When controlling for age, sex, race, ethnicity, vaccination status, and obesity in logistic regression analyses, Omicron, compared to Delta, was less likely to result in hospitalization (aOR = 0.40, [95% CI] 0.21–0.78) and any oxygen use (aOR = 0.39, 95% CI 0.17–0.87). Data were missing for race (*N* = 155), ethnicity (*N* = 202), and BMI (*n* = 243).

**TABLE 1:** Baseline characteristics and prevalence of clinical outcomes in Omicron variant vs. Delta variant infections^a^

	Omicron (*N* = 263)	Delta (*N* = 489)	Significance^b^
Baseline characteristics			
Age	37 (24–53)	42 (25–56)	*p* = 0.48
Sex (Female)	171 (65%)	256 (52%)	*p* < 0.01
Race			*p* = 0.29
*Black or African American*	34 (13%)	51 (10%)	
*White*	168 (64%)	302 (62%)	
*Other/Unknown*	61 (23%)	136 (28%)	
Ethnicity			*p* = 0.99
*Hispanic or Latino*	14 (5%)	25 (5%)	
*Not Hispanic or Latino*	178 (68%)	333 (68%)	
*Other/Unknown*	71 (27%)	131 (27%)	
Vaccinated	110 (42%)	164 (33%)	*p* = 0.02
Clinical Outcomes			
Hospital Admission	14 (5.3%)	64 (13.1%)	*p* < 0.01
Oxygen Requirement	8 (3%)	41 (8.4%)	*p* < 0.01
Mechanical Ventilation	0 (0%)	10 (2.0%)	*p* = 0.02
Death	0 (0%)	10 (2.0%)	*p* = 0.02

^a^ The data are presented as median (interquartile range) for continuous variables or number (%) for categorical variables.

^b^ Comparisons for dichotomous variables and continuous variables were calculated with the chi‐squared test (Fisher's exact test for counts <5) and rank sum test, respectively. For dichotomous variants, a relative risk with 95% confidence intervals (CI) comparing Omicron variant to Delta variant (referent) was calculated.

## 4 DISCUSSION

We evaluated the severity of disease caused by Delta and Omicron variants over a brief period across the transition from Delta to Omicron dominance in our region. Potential history bias that may have obscured the relationship between variant and severity was minimized by the short period during which healthcare seeking behavior and methods of clinical testing were assumed to be largely unchanged. We selected samples from two different time periods in order to improve the likelihood of obtaining an adequate and comparable number of both Delta and Omicron variants. Samples represented the time periods at the end of Delta variant dominance (>80% of all infections) and immediately following Omicron variant dominance (>80% of all infections), a transition that took approximately 2 weeks according to data from the CDC.

By sequencing samples of positive SARS‐CoV‐2 tests and associating these with clinical data extracted from the electronic health records, we found that infections caused by the Omicron variant were less clinically severe than those caused by the Delta variant. More patients infected with Delta (13.1%) were admitted to the hospital when compared to those infected with Omicron (5.3%), and more Delta variant infections (8.4%) required supplemental oxygen than Omicron variant infections (3.0%). Among those patients with Omicron infections, none required invasive mechanical ventilation (IMV), while approximately 2% of patients infected with Delta required IMV. Similarly, there were no recorded deaths of patients infected with Omicron, while 2% of those infected with Delta died during admission to the hospital. Among admitted patients, those with Omicron variant had lower WHO COVID‐19 Clinical Progression Scale severity categories than those with Delta (Figure 1A). Our findings are consistent with those in other recent large studies, which have found similar reductions in hospitalization, requirement for mechanical ventilation, and death associated with Omicron infection.^12^ They also agree with findings from other recent studies that suggest that severity of disease among inpatients is lower with Omicron compared to Alpha and Delta variants.^12^, ^13^

Our study had limitations. Our cohort was selected from patients seeking care in a single health system, limiting generalizability of results. Sample size was small compared to similar studies; however, the short period over which the samples were obtained minimized any divergence in healthcare seeking behavior and testing methods between the two populations and minimized increases in vaccination rates over time. We also included all positive tests, which did not account for asymptomatic testing, such as pre‐procedural testing. Furthermore, our method to detect vaccination status may underestimate true vaccination status, although we expect under‐reporting is likely similar between groups. Finally, our period of evaluation was short and does not account for the possibility that Omicron severity may change over time. Despite these limitations, our findings add to the growing literature studying the impact of SARS‐CoV‐2, especially Omicron and Delta variants on COVID‐19 morbidity and mortality in humans.

## 5 CONCLUSIONS

In a population who tested positive for SARS‐CoV‐2 over a short period surrounding the transition of dominance from the Delta to the Omicron variant, those who were infected with Omicron variant had fewer admissions, fewer requirements for oxygen and mechanical ventilation, and fewer deaths than those infected with the Delta variant as determined by whole genome sequencing. The Omicron variant appears to cause significantly less severe disease when compared to the Delta variant.

## CONFLICT OF INTEREST

All authors declare no conflict of interest to share.

## AUTHOR CONTRIBUTIONS

**Jesse O. Wrenn:** Conceptualization; data curation; formal analysis; investigation. **Suman B. Pakala:** Data curation; formal analysis; software. **Grant Vestal:** Data curation; formal analysis; methodology. **Meghan H. Shilts:** Data curation; formal analysis. **Hunter M. Brown:** Methodology; resources. **Sara M. Bowen:** Investigation; methodology; resources. **Britton A. Strickland:** Methodology. **Timothy Williams:** Methodology; resources. **Simon A. Mallal:** Conceptualization; funding acquisition; supervision. **Ian D. Jones:** Conceptualization; validation. **Jonathan E. Schmitz:** Conceptualization; funding acquisition; project administration; resources. **Wesley H. Self:** Conceptualization; formal analysis; investigation.

### PEER REVIEW

The peer review history for this article is available at https://publons.com/publon/10.1111/irv.12982.

## Supporting information


**Supporting Information S1.** Accession numbers

## Data Availability

The Global Initiative on Sharing Avian Influenza Data (GISAID) accession numbers for the whole viral genomes are available in the supporting information.
